# Intraoperative Molecular Imaging for *ex vivo* Assessment of Peripheral Margins in Oral Squamous Cell Carcinoma

**DOI:** 10.3389/fonc.2019.01476

**Published:** 2020-01-10

**Authors:** Shayan Fakurnejad, Giri Krishnan, Stan van Keulen, Naoki Nishio, Andrew C. Birkeland, Fred M. Baik, Michael J. Kaplan, A. Dimitrios Colevas, Nynke S. van den Berg, Eben L. Rosenthal, Brock A. Martin

**Affiliations:** ^1^Department of Otolaryngology – Head and Neck Surgery, Stanford University School of Medicine, Stanford, CA, United States; ^2^The Department of Otorhinolaryngology, Head and Neck Surgery, The University of Adelaide, Woodville South, SA, Australia; ^3^Department of Oral and Maxillofacial Surgery, Amsterdam University Medical Center, Amsterdam, Netherlands; ^4^Division of Medical Oncology, Department of Medicine, Stanford University School of Medicine, Stanford, CA, United States; ^5^Department of Pathology, Stanford University School of Medicine, Stanford, CA, United States

**Keywords:** near-infrared, fluorescence imaging, molecular imaging, margins, head and neck cancer, oral cavity, antibody

## Abstract

**Objective:** Complete surgical resection is the standard of care for treatment of oral cancer although the positive margin rate remains 15–30%. Tissue sampling from the resected specimen and from the wound bed for frozen section analysis (FSA) remains the mainstay for intraoperative margin assessment but is subject to sampling error and can require the processing of multiple samples. We sought to understand if an *ex vivo* imaging strategy using a tumor-targeted fluorescently labeled antibody could accurately identify the closest peripheral margin on the mucosal surface of resected tumor specimen, so that this “sentinel margin” could be used to guide pathological sampling.

**Materials and Methods:** Twenty-nine patients with oral squamous cell carcinoma scheduled for surgical resection were consented for the study and received systemic administration of a tumor-targeted fluorescently labeled antibody (Panitumumab IRDye800CW). After surgical resection, the tumor specimen was imaged using a closed-field fluorescent imaging device. Relevant pathological data was available for five patients on retrospective review. For each of these five patients, two regions of highest fluorescence intensity at the peripheral margin and one region of lowest fluorescence intensity were identified, and results were correlated with histology to determine if the region of highest fluorescence intensity along the mucosal margin (i.e., the sentinel margin) was truly the closest margin.

**Results:** Imaging acquisition of the mucosal surface of the specimen immediately after surgery took 30 s. In all of the specimens, the region of highest fluorescence at the specimen edge had a significantly smaller margin distance than other sampled regions. The average margin distance at the closest, “sentinel,” margin was 3.2 mm compared to a margin distance of 8.0 mm at other regions (*p* < 0.0001).

**Conclusions:** This proof-of-concept study suggests that, when combined with routine FSA, *ex vivo* fluorescent specimen imaging can be used to identify the closest surgical margin on the specimen. This approach may reduce sampling error of intraoperative evaluation, which should ultimately improve the ability of the surgeon to identify the sentinel margin. This rapid sentinel margin identification improves the surgeon's orientation to areas most likely to be positive in the surgical wound bed and may expedite pathology workflow.

## Introduction

Surgical resection with curative intent remains a mainstay in the treatment of solid tumors. Patient outcomes are largely dependent on obtaining clear surgical margins, as locoregional recurrence rates are significantly higher when residual disease exists at or near the margin (1). Unfortunately, the rates of positive margins in most branches of surgical oncology have remained stagnant over the past 15 years (2). This has been a particular burden in the management of head and neck cancers, with positive margin rates ranging from 15–30% (3).

To obtain a tumor-negative margin in head and neck cancer, the surgeon must attain a 5-mm margin of normal tissue around the tumor, based on extensive survival data demonstrating that smaller margins result in worse survival (4). To accurately measure this margin of normal tissue, the margin should be assessed on the specimen rather than the wound bed, although this remains controversial (5, 6). Obtaining a consistent 5-mm cuff of normal tissue is challenging since surgeons largely rely on visual and tactile cues when operating. While many novel technologies have emerged to assist in delineation of margins, none have been incorporated into the standard surgical and pathological workflow (7). Therefore, currently the standard of care for intraoperative margin assessment is the use of frozen section analysis (FSA). Here, the specimen margins are sampled by the surgeon and/or pathologist for immediate processing and evaluation in parallel to surgery. Results are communicated back to the surgeon so that further resection can be performed if required.

There are two critical limitations with this current practice of identifying positive margins. The first limitation is the fact that sampling of the tumor margin, whether by the surgeon or by the pathologist, is subject to error. Most specimens are 5–10 cm in diameter, and only a fraction of the margin can be sampled; therefore, the likelihood of a false negative assessment is high. The second limitation is that following resection, the tumor specimen must leave the operating room, and the orientation of the specimen relative to the wound bed is often lost. Consequently, when the pathologist reports the FSA results to the operating room, it is challenging for the surgeon to correlate where in the patient the positive margin was identified.

A number of novel imaging technologies have been utilized in surgical oncology and have been met with variable success. Narrow band imaging (NBI) has been available for many years and has been used for both early detection and screening of head and neck cancer, as well as for intraoperative margin assessment (8, 9). The technology relies on the detection of hemoglobin, which in turn allows for enhanced visualization of neoangiogenesis, a known phenomenon in solid tumors (10). However, the technique is challenging to master, and is heavily reliant on the subjective interpretation of the images. Furthermore, the technique is influenced heavily by tissue properties and modified vascularity, which are often seen with tumors of the head and neck (11). Another emerging technology for intraoperative margin analysis during oncological surgery is the use of fluorescence molecular imaging (12–14). Fluorescently labeled antibodies allow for highly specific targeting of cancer cells and can be utilized for a myriad of imaging techniques. Leveraging this technology in the current study, we propose a novel methodology for rapid, objective, and reproducible identification of the closest margin on the peripheral mucosal surface of the resected tumor specimen, termed the “sentinel margin.” We have previously demonstrated that the sentinel margin strategy can be applied to evaluate the deep surface of the surgical specimen, and here we focus on the mucosal margin (15).

Successful validation of the proposed fluorescent imaging-based specimen mapping technique would allow for more accurate sampling for FSA from the tumor specimen. This would lead to improved accuracy of intraoperative tumor margin analysis and ultimately improve patient prognosis. Furthermore, by targeting the sentinel margin for FSA, fewer samples would be required to adequately assess the entire peripheral margin, with secondary benefits such as a significantly reduced burden on the pathologist and fewer delays in operating time.

The objective of this retrospective proof-of-concept study was to determine if the “sentinel margin” as identified by our proposed fluorescent imaging-based specimen mapping technique could accurately identify the closest surgical margin at the peripheral, mucosal border in order to improve accuracy of FSA sampling and to improve surgical orientation to the wound bed when further resection is required.

## Materials and Methods

### Study Design

A Phase I study evaluating panitumumab-IRDye800CW was approved by the Stanford Institutional Review Board (IRB-35064; NCT02415881). The study process, safety of panitumumab-IRDye800CW, and pharmacokinetics of the drug have been previously reported (16). Consented patients were infused 1–5 days prior to surgery with a 50 mg dose of panitumumab-IRDye800CW. Following primary tumor resection, the mucosal surface of the tumor specimen was imaged in a closed-field fluorescence-imaging device (PEARL Trilogy, LI-COR Biosciences Inc., Lincoln, NE). The specimen was then sent to pathology for standard-of-care histological assessment. Specimens were formalin-fixed overnight and serially cross-sectioned at 5 mm intervals. These cross-sections were then further divided as necessary to fit in cassettes for paraffin embedding, after which a representative 5 μm section was cut from each paraffin block for routine hematoxylin and eosin (H&E) staining. Histopathological assessment was performed by a board-certified pathologist who outlined regions of squamous cell carcinoma on the slide. The slides were then digitized (NanoZoomer 2.0-RS; Hamamatsu Photonics K.K., Hamamatsu, Japan) and analyzed for study purposes. Included in the current retrospective study were patients with oral cavity squamous cell carcinoma whose primary tumor had less than a 1 cm depth of invasion and no cortical bone involvement. These strict inclusion criteria were applied to ensure patient specimens were amenable to the rigorous retrospective histological analysis as described below. Therefore, 5 patients were included in this proof-of-concept study.

### Fluorescent Imaging Based Specimen Mapping

**Figure 1:**
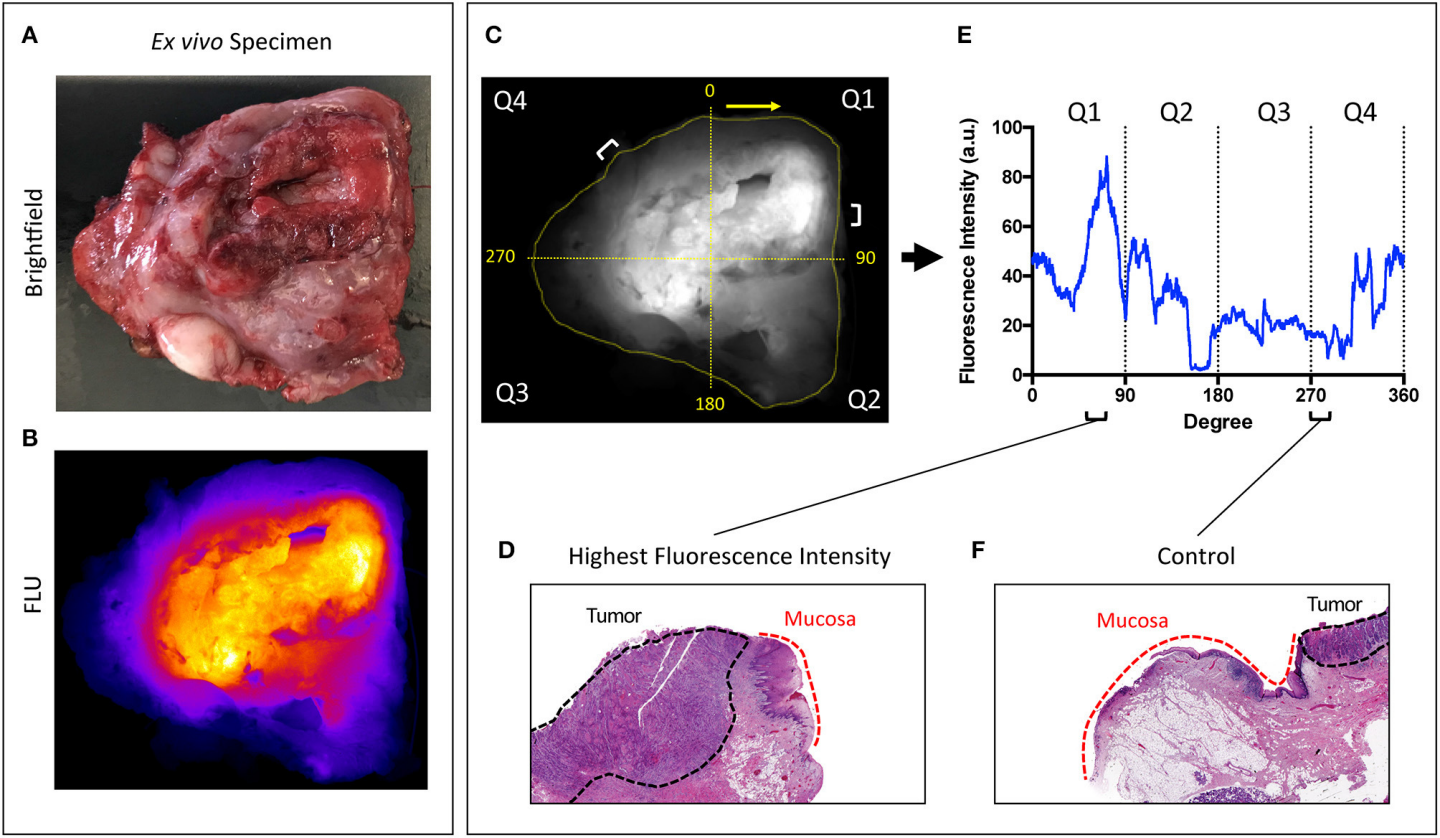
Overview of workflow. Representative brightfield **(A)** and closed-field fluorescence image **(B)** of a resected specimen. **(C)** Fluorescent image with mask applied circumferentially around tumor margin. Specimen divided into 4 quadrants labeled Q1−4 clockwise from 0 degrees. **(D,F)** H&E slides taken from regions of highest fluorescence intensity and control region of low fluorescence intensity with tumor and normal mucosa delineated. **(E)** Graph illustrating corresponding fluorescence intensities to peak and control at location on circumferential mask.

The brightfield and fluorescence images obtained from the closed-field imaging device were loaded into ImageJ (version 1.50i, National Institute of Health, Washington D.C., ML). Using the brightfield image of the primary tumor specimens, a mask was manually created along the periphery of the specimen, ~1 mm within the edge to avoid any potential for edge artifact during fluorescence imaging. This mask was then applied onto the fluorescence image obtained from the closed-field imager, allowing for measurement of the fluorescence signal along the length of the mask. The raw fluorescence data was analyzed in an 8-bit grayscale format with black as 0 and white as 255. A graphical representation of the workflow can be found in Figure 1.

As the specimens were processed according to current standard-of-care for gross histological assessment, only a portion of the periphery was retrospectively analyzable with available perpendicular sections of tumor to peripheral margin. From this analyzable portion of the specimen, two regions of highest fluorescence intensity were selected, as well as one region of lowest fluorescence. Careful annotation of brightfield images taken throughout each stage of gross specimen processing allowed for a direct correlation of specimen fluorescence in the regions of interest with corresponding microscopic histology.

To decrease interference of interpatient variables such as dose, infusion-to-surgery window, epidermal growth factor receptor (EGFR)-expression and other biological factors, patients were used as their own internal control by comparing high fluorescence regions to low fluorescence regions on the same specimen as was previously described and validated in our deep sentinel margin mapping study (15).

### Correlation of Fluorescence Signal With Margin Distance

Along the periphery of the specimen, for each of the two regions of the highest fluorescence intensity and for one region of low intensity (which served as a control), the margin distance was measured. The margin distance was defined as the distance in millimeters between the tumor edge and the specimen edge (i.e., the surgical cut) on the H&E-stained microscopic sections. First, the margin distance at the region of highest fluorescence within the analyzable domain was compared to the margin distance at the lowest fluorescence intensity region. Second, to determine if the margin distance correlated with the fluorescence signal, the margin distance at the highest fluorescence intensity region was compared to the second highest fluorescence intensity region.

In order to register the microscopic findings to the fluorescence signal on the intact specimen, the specimen was virtually reconstructed from the 5 mm thick macroscopic cross-sections. This process has been described previously (17, 18). As the cross-sections are ~5 mm thick with each submitted for microscopic evaluation, the margin of error for mapping histologic findings to points along the mucosal edge of the intact specimen is <5 mm; this margin of error does not influence the margin distances which are measured in perpendicular planes. On each histological section, the margin distance was measured using ImageJ (US NIH, Bethesda MD, USA) three independent times and then averaged.

### Statistical Analysis

Data was imported into GraphPad, Version 8.0c (La Jolla, California, USA), and the intra-specimen comparison of margin distance was done using the Wilcoxon-signed-ranked test. *P* < 0.05 were considered statistically significant.

## Results

### Subjects

Between December 2015 and June 2018 a total of 29 patients underwent infusion of panitumumab-IRDye800 for intraoperative fluorescent imaging including *ex vivo* fluorescence imaging of their tumor specimen directly after resection. Of these patients, only five had sufficient pathological data to be included in the study. Patient and tumor characteristics are presented in Table 1. Imaging acquisition of the peripheral surface of the specimen took ~30 s, after which the specimen was sent to pathology and processed for standard of care assessment. As part of the retrospective analysis, the sentinel margin was identified by determining the region of highest fluorescence intensity along the specimen edge. Each serial cross-section of the specimen was also assessed by fluorescence imaging, and the sentinel margin distance was compared to all the other margin distances with low fluorescence signal obtained in the tissue sections (~8–18 analyzable margins per specimen). We chose to evaluate two margins as potential sentinel margins (where the fluorescence was highest and second highest at the specimen edge).

**Table 1:** Patient and tumor characteristics.

**#**	**Age**	**Sex**	**Tumor** ** site**	**Tumor** ** stage**	**Tumor** ** grade**	**Smoking**	**Alcohol**	**LVI**
1	62	M	Buccal	T2N0Mx	II	N	N	N
2	46	M	Lateral tongue	T1N0Mx	I–II	Y	Y	N
3	69	F	Buccal	T1N0Mx	I–II	N	N	N
4	65	F	Buccal	T2N2bMx	II	Y	Y	N
5	70	F	Buccal	T3N0Mx	I	N	Y	N

*Tumor stage was the pathologic staging, and tumor grade was the histologic grading (I: well-differentiated, II: moderately-differentiated, III: poorly-differentiated). Smoking and alcohol use were considered “yes” if the patient had a prior history or was an active user. LVI: lymphovascular invasion. N, No; Y, Yes; Unk, unknown status*.

### High Fluorescence Intensity Regions (Sentinel Margin) vs. Low Fluorescence Intensity Regions (Controls)

**Figure 2:**
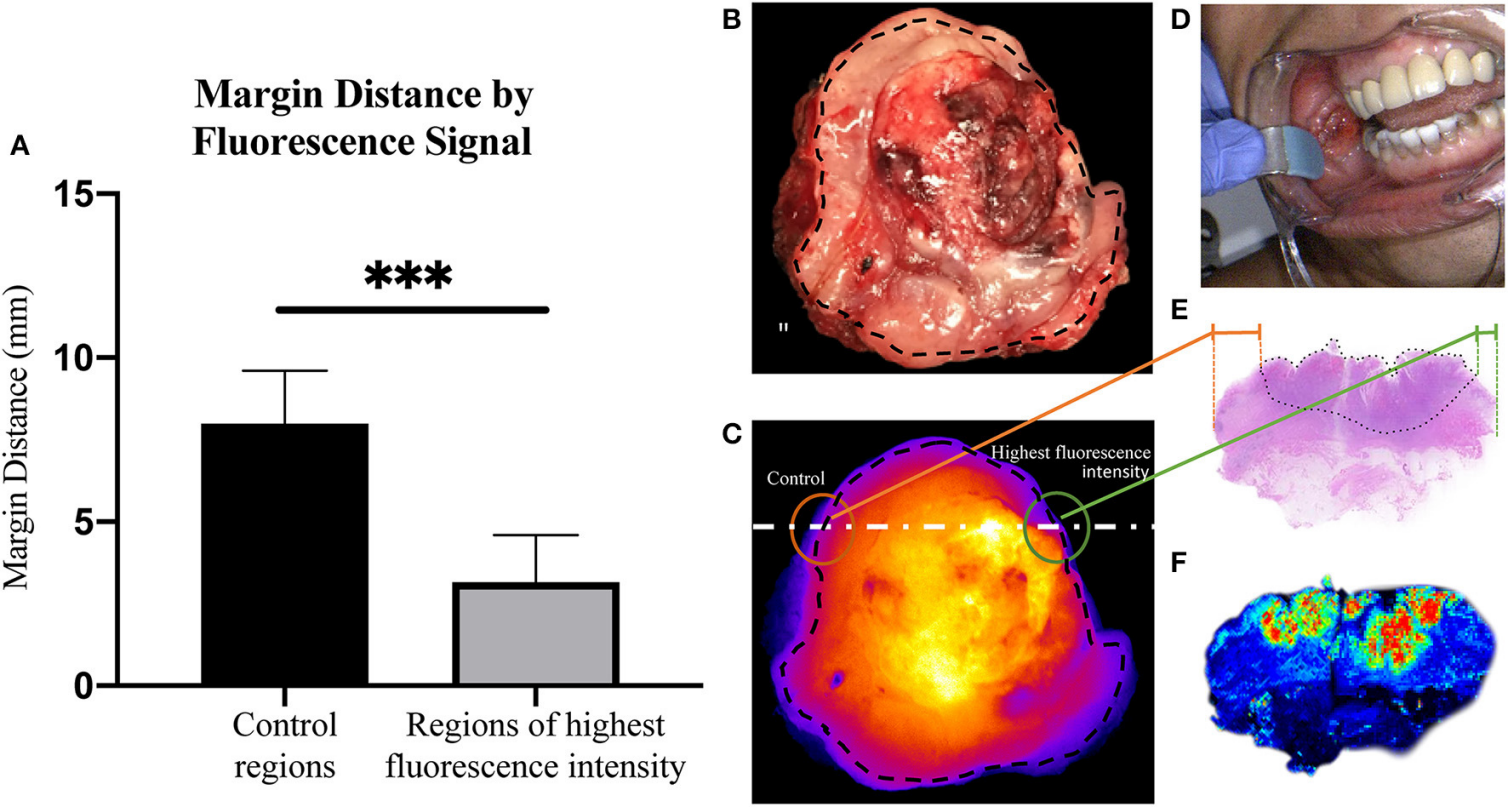
Margin distance by fluorescent signal. **(A)** Graph showing increase in margin distance at control regions when compared to sampled regions of highest fluorescence intensity. Representative brightfield image of resected tumor specimen **(B)** taken from buccal region in patient, seen in **(D)**. **(C)** Corresponding closed-field fluorescent image of resected tumor specimen with black dotted line indicating overlaid circumferential mask, white dashed line indicating slice from which H&E slide **(E)** was taken, highlighting the difference in margin distance at the periphery between control region and region of highest fluorescence intensity. **(F)** High resolution image taken from Odyssey demonstrating fluorescence distribution within microscopic section. ***p < 0.0001.

From each primary tumor specimen, two sentinel margins were identified by determining the regions of highest fluorescence at the cut mucosal surface of the specimen. The margin distance at the sentinel margin was compared to the margin distance at other sites with low fluorescence. In all specimens (100%), as shown in Figure 2, the margin distances at the sentinel margins were significantly lower than the margin distances at other regions; the average margin distance at the sentinel margins was 3.2 mm compared to 8.0 mm in other regions evaluated (*p* < 0.0001).

### Comparison of Margin Distances at the Fluorescence Extremes

**Figure 3:**
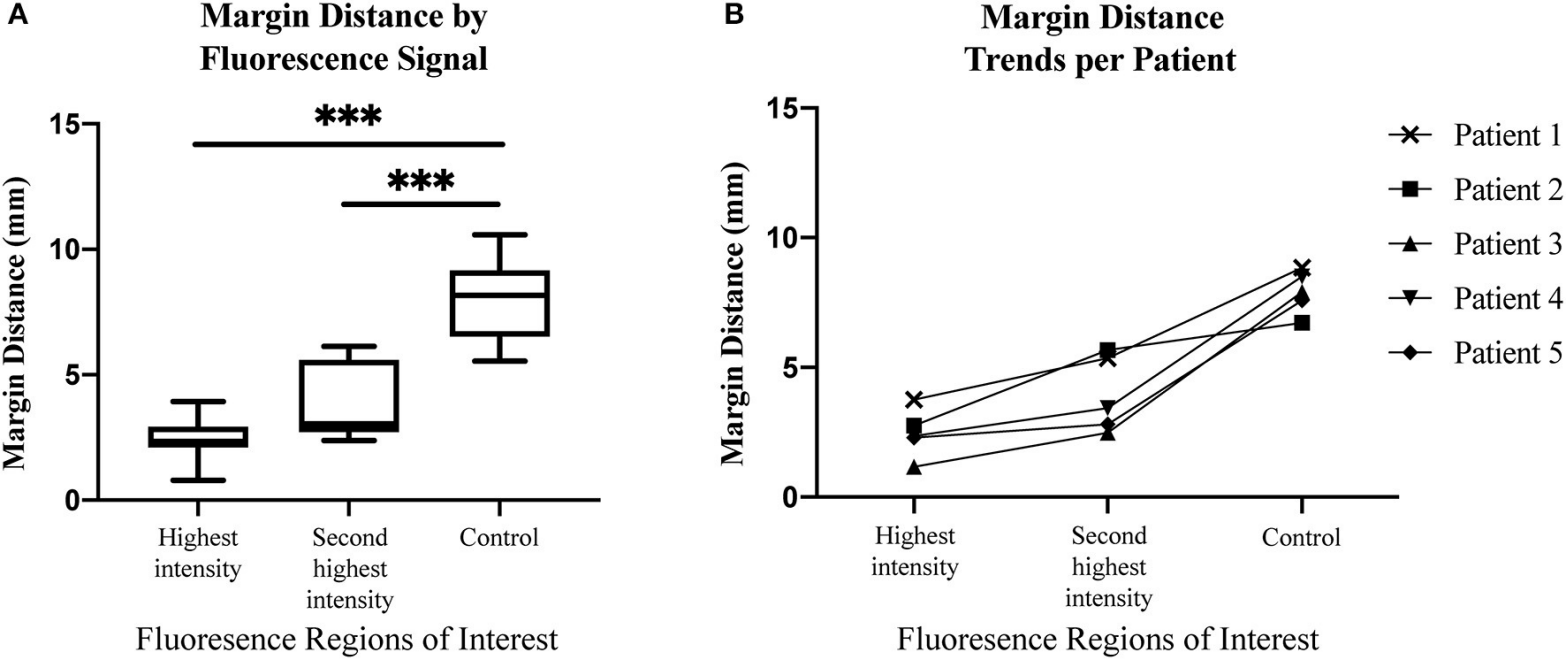
**(A)** Box and whisker plots demonstrating margin distance by fluorescent signal. **(B)** Graph demonstrating margin distance trends from region of highest fluorescence intensity to second highest fluorescence intensity, to control region per patient. ***p < 0.0001.

Next, we sought to determine if margin distance would increase linearly in the regions of highest to lowest fluorescence intensity along the periphery of the mucosal surface. A significant difference was found for margin distance when comparing each group (first sentinel margin, second sentinel margin, and low-fluorescence control). The sentinel margin (highest fluorescence region at the cut edge of the specimen) measured on average 2.4 mm, compared to 4.0 mm for the second sentinel margin and 8.0 mm for control regions (*p* < 0.0001). As shown in Figure 3, in all the imaged specimens, the margin distance was closest at the point of highest fluorescence signal, the sentinel margin, compared to the second, with the largest margin at the low fluorescence intensity region. The average increase in margin distance when comparing the first and the second sentinel margins was 1.5 ± 0.90 mm. Importantly, the fluorescence intensity also accurately predicted the closest margin distances when correlated with final standard-of-care histopathologic assessments by H&E staining.

## Discussion

The present study demonstrates that after systemic administration of a targeted fluorescent agent, resected oral tumor specimens can be quickly imaged to determine the closest or “sentinel” margin on the peripheral mucosal surface. This proof-of-concept study has two important clinical implications for future specimen analysis in near real-time during surgery. First, it will reduce sampling error when selecting tissue for FSA from the primary specimen. Second, by generating an immediate intraoperative image available to the surgeon and pathologist, it improves the surgeon's ability to remain oriented to which areas are sampled for FSA and aids with the accurate, targeted re-resection from the wound bed if required.

The proposed approach has previously been described by our team for targeting the closest tumor margin on the deep surface (15). The term “sentinel margin” was first introduced to designate the closest margin, which may or may not be positive but will be the margin most at risk. If the sentinel margin is identified as negative (>5 mm) on FSA, one could reliably predict that the rest of the margins from other areas of the tumor specimen would also be negative in the current study. Accurately selecting margins that are most at risk for being close and/or positive on FSA has potential to not only decrease the burden on the pathologist, but also to shorten the surgical procedure time. In the case where the sampled margin returns positive for carcinoma within 5 mm of the cut edge, the surgeon can resect additional tissue and repeat the FSA procedure until the margin is clear. In these instances, because tumor specimen imaging takes place on the back table in the operating room, in parallel with the operation, the surgeon can assess the fluorescence image from re-resected tissue in near-real time.

The other potential contribution of this technology to the surgical workflow is the opportunity to perform the initial assessment immediately after removal of the specimen so that the surgeon can remain oriented to the wound bed. Once the sentinel margin is identified, the surgeon can confirm the corresponding area in the wound bed and then send the specimen for pathological determination by FSA. Fluorescence images can then also be made available to the pathologist, allowing for more direct and accurate communication of the margins at risk and those sampled by FSA.

This proposed technique is built upon the knowledge that 90% of squamous cell carcinomas in the head and neck have upregulated EGFR (19). Antibody-based contrast agents, such as panitumumab-IRDye800CW, leverage this fact and strongly and specifically bind tumor cells with higher affinity than adjacent, healthy tissue. This allows for a robust imaging technique that can detect regions of tissue harboring cancer. Specificity for this antibody-dye bioconjugate for its receptor has been thoroughly studied, and previous studies have demonstrated excellent specificity for EGFR (20, 21). Although demonstrated for EGFR, this proposed technique can be used for margin assessment to any highly specific targeted-imaging agents, provided the expression of the target in tumor tissue is vastly different from that of normal tissue. Furthermore, since this technique relies on using relative fluorescence intensity differences where each patient serves as their own control, the methodology is not influenced by differences in infusion time or dosing. It is known that fluorescence imaging techniques have suffered from limitations with tissue auto-fluorescence. Fluorescent dyes in the near-infrared range of the light spectrum do not suffer from these limitations to any reasonable extent, allowing for improved contrast, and deeper penetration. Depths up to around 6 mm have been reported with IRDye800CW, which is fortuitous as positive surgical margins in the head and neck are 5 mm or less (5). Breast and skin cancer are two other cancer types that leverage these facts and may also be amenable to the proposed intraoperative imaging-based specimen mapping technique.

A limitation of this study is its retrospective nature and therefore the low number of patients that could be included. Because accurate assessment of the margin distance requires taking a perpendicular section of tumor to the closest (sentinel) margin, microscopic assessment of the relevant fluorescent area was possible only in select cases retrospectively. Nevertheless, we feel that since each specimen had numerous peripheral margins analyzed, the current data is sufficient to ensure the robustness of this methodology. However, in order to further evaluate the clinical efficacy of this technique, a larger prospective trial is warranted where sentinel peripheral margins highlighted fluorescently on the back table are correlated to clinical suspicion prior to undergoing selective FSA as appropriate. Such a trial investigating the accuracy of sentinel deep and peripheral margins using this technology is currently underway at our institution. This technique, once validated on a larger series of specimen, could join other techniques, such as NBI, aimed at improving margin control.

## Conclusion

This retrospective, proof-of-concept study demonstrates that fluorescence molecular imaging can be used to detect regions on the periphery of the resected tumor specimen that correlate with the closest mucosal margin, the “sentinel margin.” The clinical application of this specimen mapping technique to surgical management would allow identification of the sentinel margin for more accurate and efficient intraoperative sampling. Fluorescence-guided FSA could thus reduce diagnostic error secondary to specimen sampling and expedite the pathology workflow. When additional resection is required following FSA, near real-time fluorescent imaging can facilitate improved communication of positive or close margins between the surgeon and pathologist by maintaining orientation of the specimen to the wound bed and aiding with completeness of resection. With these benefits in mind, this *ex vivo* near real-time imaging strategy has great potential to ultimately improve margin control rates in oncological head and neck surgery.

## Data Availability Statement

The datasets generated for this study are available on request to the corresponding author.

## Ethics Statement

The studies involving human participants were reviewed and approved by Stanford Institutional Review Board. The patients/participants provided their written informed consent to participate in this study.

## Author Contributions

Data collected and analyzed by SF, GK, SK, NN, and NB. Manuscript prepared by SF, GK, and SK. Study conception and designed by NB, AB, FB, AC, ER, and BM. Manuscript edited by all authors.

### Conflict of Interest

The authors declare that the research was conducted in the absence of any commercial or financial relationships that could be construed as a potential conflict of interest.
